# Anti-Freezing, Non-Drying, Localized Stiffening, and Shape-Morphing Organohydrogels

**DOI:** 10.3390/gels8060331

**Published:** 2022-05-25

**Authors:** Jiayan Shen, Shutong Du, Ziyao Xu, Tiansheng Gan, Stephan Handschuh-Wang, Xueli Zhang

**Affiliations:** College of Chemistry and Environmental Engineering, Shenzhen University, Shenzhen 518060, China; 2170225620@email.szu.edu.cn (J.S.); 2060221013@email.szu.edu.cn (S.D.); 1910223058@email.szu.edu.cn (Z.X.); gantiansheng@szu.edu.cn (T.G.); stephan@szu.edu.cn (S.H.-W.)

**Keywords:** organohydrogels, anti-freezing, shape-morphing, tough hydrogels, flexible electronics

## Abstract

Artificial shape-morphing hydrogels are emerging toward various applications, spanning from electronic skins to healthcare. However, the low freezing and drying tolerance of hydrogels hinder their practical applications in challenging environments, such as subzero temperatures and arid conditions. Herein, we report on a shape-morphing system of tough organohydrogels enabled by the spatially encoded rigid structures and its applications in conformal packaging of “island–bridge” stretchable electronics. To validate this method, programmable shape morphing of Fe (III) ion-stiffened Ca-alginate/polyacrylamide (PAAm) tough organohydrogels down to −50 °C, with long-term preservation of their 3D shapes at arid or even vacuum conditions, was successfully demonstrated, respectively. To further illustrate the potency of this approach, the as-made organohydrogels were employed as a material for the conformal packaging of non-stretchable rigid electronic components and highly stretchable liquid metal (galinstan) conductors, forming a so-called “island–bridge” stretchable circuit. The conformal packaging well addresses the mechanical mismatch between components with different elastic moduli. As such, the as-made stretchable shape-morphing device exhibits a remarkably high mechanical durability that can withstand strains as high as 1000% and possesses long-term stability required for applications under challenging conditions.

## 1. Introduction

Natural living systems have organs that can deform and exhibit different shapes to carry out their biological roles even under challenging environments, e.g., subzero temperatures and dry environments. To mimic natural systems, a large number of artificial shape-morphing materials and devices have been developed for numerous applications in human–machine interfaces, soft robotics, and healthcare [1,2,3,4,5,6,7,8,9]. Hydrogels [10], particularly tough hydrogels, with abundant properties and functionalities, are anticipated to be ideal for artificial shape morphing due to their intriguing mechanical properties, e.g., high stretchability and toughness [11,12,13,14,15,16,17]. Various anisotropic elements can be encoded into the hydrogels and can be triggered to guide the shape morphing by exposing to different stimuli, e.g., pH, solvents, light, magnetic fields, and electric fields [18,19,20,21,22,23,24,25,26,27].

Although numerous advances in shape morphing of hydrogels were reported by the combination of state-of-the-art techniques and emerging functional materials [26,28,29,30], several critical issues remain. In terms of materials, for example, hydrogels easily freeze at subzero temperatures and lose water at low humidity [31]. The icing and drying of hydrogels cause not only the change of their shapes but are also detrimental to their functionalities, such as the dramatic change in their mechanical properties upon freezing [32]. Fortunately, the past decade has witnessed several remarkable efforts devoted to addressing the icing and drying issues [33,34,35,36,37]. For example, Zhao et al. reported a skin-inspired method by employing an elastomeric sealing layer to protect the hydrogels from dehydration [33]. Another approach is to exchange part or all of the aqueous phase with an organic solvent, exhibiting low vapor pressure. Thus, prepared gels are denoted in literature as organohydrogels (partially displaced water) or organogels (fully displaced water or made directly in organic solvent). Liu et al. used water and oil as the medium to achieve temperature-tolerant organohydrogels, with a working temperature range from −78 to 80 °C [37]. Subsequently, they applied an ethylene glycol–water binary solvent to achieve anti-freezing organohydrogels [36]. Lu et al. used a glycerol–water binary solvent to fabricate hydrogels with long-lasting weight retention over a wide temperature range, from −20 to 60 °C [34]. More recently, we developed a solvent displacement method for the swift transformation of prefabricated tough hydrogels into tough organohydrogels, relying on the displacement of water with single (or multiple) cryoprotectant(s) (e.g., glycerol, glycol) [35]. Taking into account the extremely high freezing and drying tolerance, tough organohydrogels in principle are particularly suitable for shape morphing in challenging environments, such as subzero temperatures and arid conditions. One crucial remaining challenge is to develop strategies for the encoding of anisotropic elements, which serve for programmable guiding of the shape morphing.

Anisotropic elements can be encoded by tuning the mechanical properties locally. To tune the mechanical features of functional materials, a variety approaches have been pro-posed. For example, the liquid–solid phase transition of a solvent or a dispersed phase was exploited to increase the stiffness of fibers or a composite [5,38]. More localized approaches include the near-infrared triggered strategy to tune the stiffness of a 3D hydrogel and multivalent strengthening methods, relying on the displacement of a “weak” (physically) crosslinking ion by a strong crosslinking ion [15]. For Ca-alginate/PAAm tough hydrogels, the addition of Fe^3+^ ion endowed the resulting hydrogel with exceptional stiffness [39]. Notably, such stiffened gels can be softened by reducing the Fe^3+^ to Fe^2+^, which renders this kind of ion an interesting choice for shape morphing and localized stiffening applications [40]. However, the concept of localized stiffening with Fe^3+^ ions has not been translated to organohydrogels.

Another issue of organohydrogels compared to hydrogels is the fact that organohydrogels are encumbered with low electrical conductivity. To endow the organohydrogel with conductivity, the organohydrogel approach is coupled with liquid metal (Galinstan) as a flexible and stretchable conductor. Liquid metal was chosen as it features fluidity (ca. 2 mP s), rendering it nearly infinitely bendable and stretchable, and high electrical conductivity (~3.4 × 10^4^ S/cm) [41]. Furthermore, it remains in its liquid state far below its melting temperature of 10.5 °C (Galinstan) due to supercooling, and freezing is typically observed at temperatures below −20 °C [41]. Therefore, both the liquid metal and the hydrogel are endowed with moldability at subzero temperatures, and should be an interesting approach for hydrogel soft electronics.

**Scheme 1:**
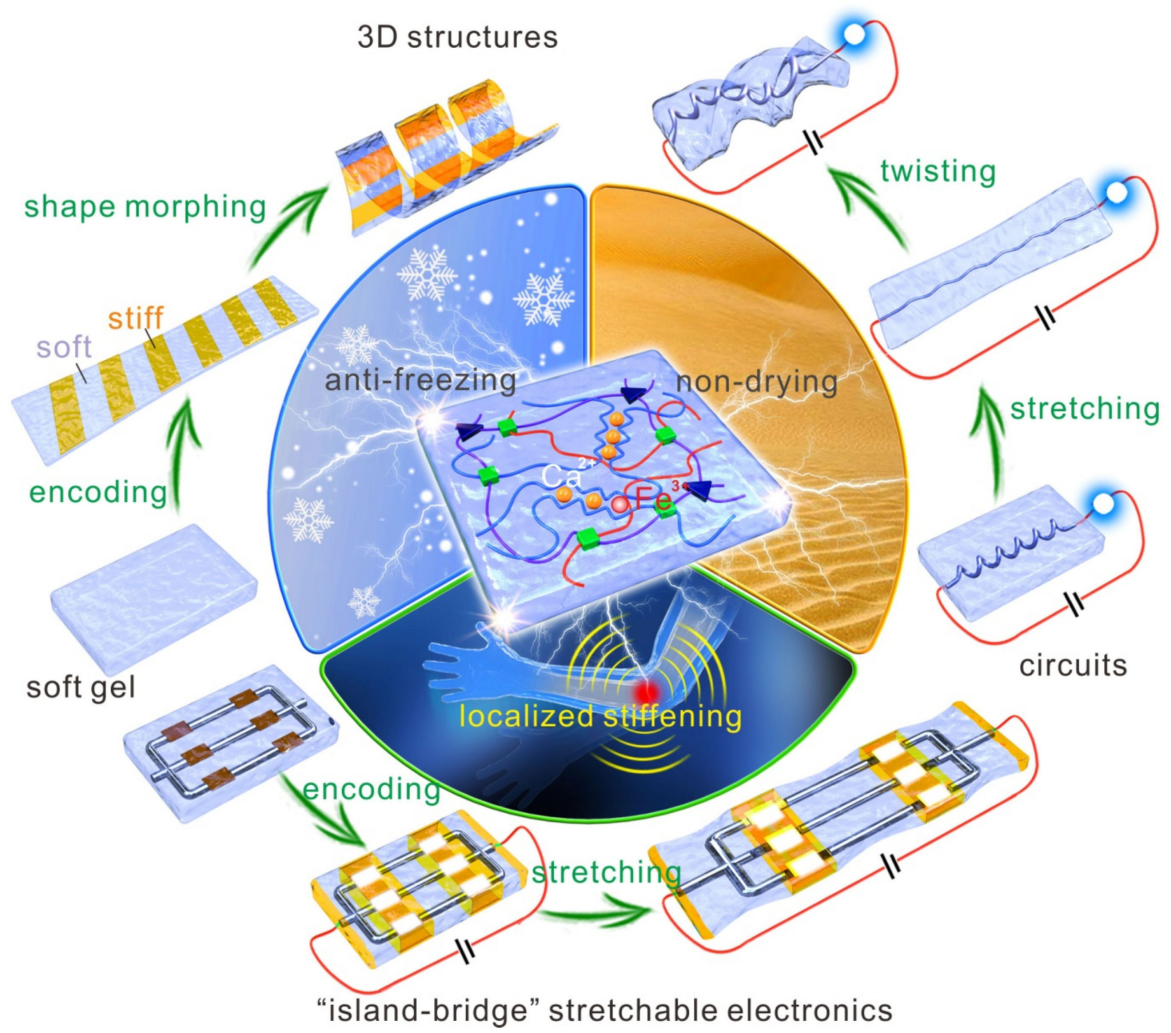
Tough organohydrogels with anti-freezing, non-drying, localized stiffening, and shape-morphing capabilities toward applications in conformal packaging of mechanically durable and stretchable electronics.

Herein, we report an anti-freezing, non-drying, shape-morphing system of tough organohydrogels enabled by spatially distributed, stiffness-tunable structures, and their applications in conformal packaging of “island–bridge” stretchable electronics. As shown in Scheme 1, three important features (anti-freezing, non-drying, and localized stiffening) are achieved simultaneously with tough organohydrogels. Hence, programmable shape morphing can be realized artificially under challenging conditions, such as subzero temperatures and long-term storage under arid conditions. Besides, conformal packaging of the rigid and soft components in one material of tough organohydrogels for stretchable electronics is enabled by the spatially distributed, stiffness-tunable feature. To validate this method, we employed glycerol-based Ca-alginate/polyacrylamide (PAAm) tough organohydrogels as a model system, which consist of a Ca (II) divalent ionically crosslinked alginate and covalent crosslinked PAAm networks. The mechanical stiffening of tough organohydrogels, in bulk or locally with Fe (III) ions, was either conducted via soaking or surface patterning of Fe (III) aqueous solutions. The shape morphing of organohydrogels was demonstrated at subzero temperatures by releasing the stress applied to a pre-stretched gel. As proof-of-concept, the as-made tough organohydrogels were applied as a material with locally tunable stiffness for conformal packaging of electronic components with different stiffness, i.e., arrays of stiff light-emitting diode (LED) lamps and soft conductors of room-temperature liquid metals (galinstan), forming so-called “island–bridge” structures. To further evaluate the durability and stability, fatigue tests and long-term observation were conducted.

## 2. Results and Discussion

The structure of the DN hydrogel (Ca-alginate/PAAm) is given in the Supplementary Materials (Figure S1). The structure of the DN hydrogel and the organohydrogel was corroborated by Fourier transform infrared spectroscopy, as shown in Figure S2. The spectra signify the establishment of the double network hydrogel and the displacement of water with glycerol.

We first demonstrated the stiffening of glycerol-based Ca-alginate/PAAm tough organohydrogels in bulk by a multivalent ionic stiffening strategy. Traditional hydrogels can be strengthened by applying a number of approaches, including double network, physical interactions, etc. For organohydrogels, however, limited examples of mechanical strengthening have been reported [42]. In the present study, the stiffening of the organohydrogels is realized by exposing the organohydrogel to Fe (III) aqueous solutions. In the experiment, we systematically investigated the stiffening effect of Fe (III) ion aqueous solution (1 mol/L) with different soaking times, ranging from 0 to 60 min. Indeed, a dramatic increase in the elastic modulus from 40 kPa to ~700 kPa is observed (Figure S3). The stiffening of the gel is attributed to the formation of trivalent ionically crosslinked centers between Fe (III) ions and alginate chains. A similar finding was also observed in Ca-alginate/PAAm tough hydrogels [43]. Interestingly, the elastic modulus increases when the Fe^3+^-treated organohydrogel is exposed to an environment with relative humidity higher than 50% while the maximum sustainable stress decreases, as shown in Figure S4. Although a longer soaking time leads to a slightly higher elastic modulus, a shorter time, such as 1–5 min (or surface patterning with small amounts of aqueous solutions), is preferred to minimize the effect of solvent displacement. Longer soaking times lead to an unfavorable displacement of glycerol with water, which is detrimental to the freezing and drying tolerance. The FTIR spectrum of the stiffened organohydrogel is very similar to the organohydrogel. The big difference between these two spectra is that the bands 1670 cm^−1^ and 1610 cm^−1^ (C=O and COO^−^ groups of the alginate) dwindle upon addition of iron (III), likely due to association with it. The homogeneous distribution of the iron ion in the gel is illustrated in Figure S5 (energy dispersive X-ray spectroscopy, EDS).

**Figure 1:**
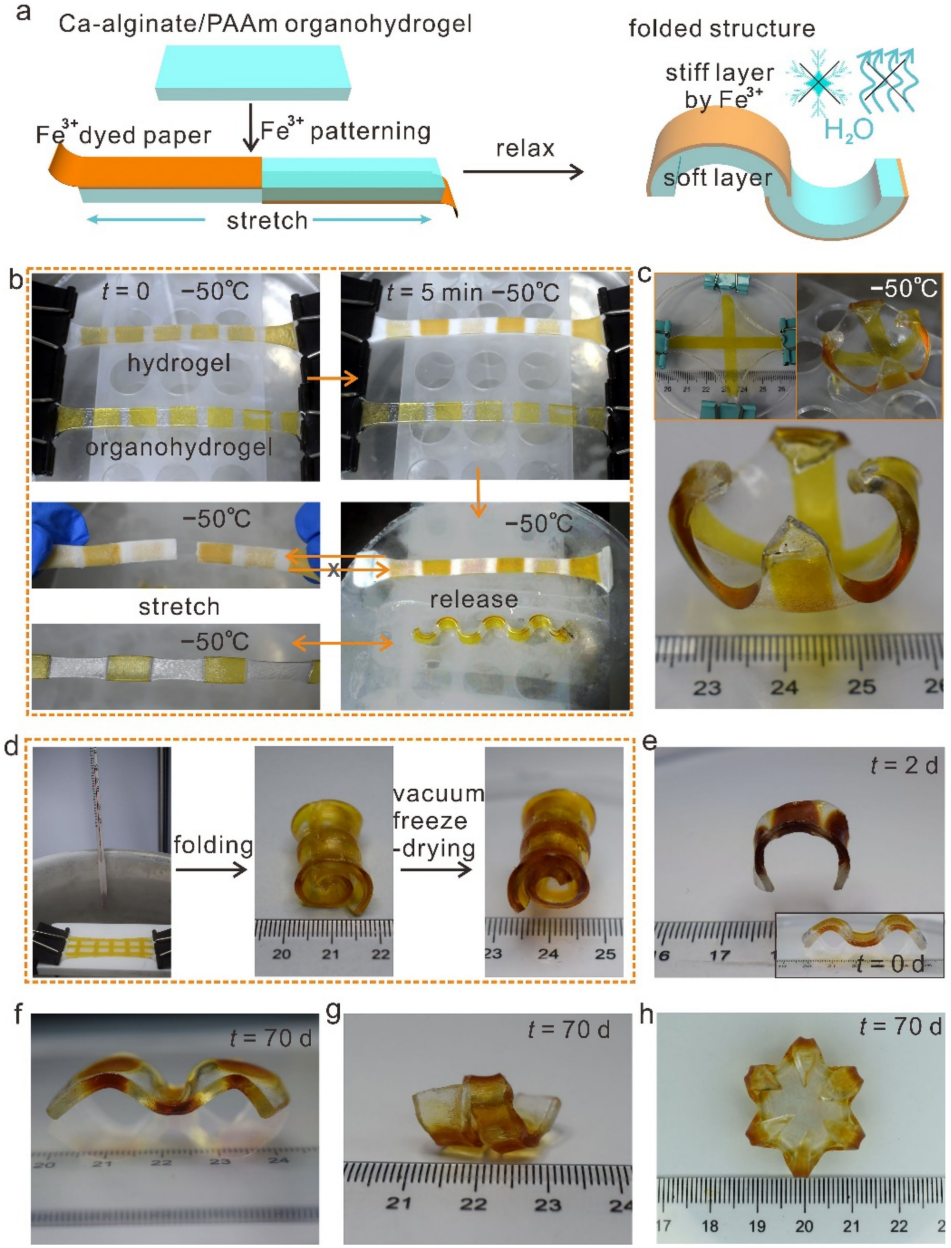
Shape morphing of Fe (III)-patterned tough organohydrogels at −50 °C and long-term storage under arid conditions. (**a**) Scheme of the shape morphing process enabled by the paper transfer patterning of Fe(III) ion aqueous solution on a pre-stretched tough organohydrogel. (**b**) Pre-stretched patterned gels (organohydrogel and hydrogel) were subjected to a temperature of −50 °C. After release of the tensile strain, the organohydrogel folded into a sinuous line, while the frozen hydrogel remained in its stretched shape. (**c**) A more complex folded flower shape realized at −50 °C. (**d**) Illustration of the high drying tolerance of the organohydrogel under vacuum freeze-drying conditions. (**e**) Dried hydrogel after exposure to an arid environment (50% humidity) for 2 days. (**f**–**h**) Soft organohydrogels after exposure to an arid environment (50% humidity) for 70 days: (**f**) sinuous line shape, (**g**) spiral shape, and (**h**) flower shape.

The binding process between multivalent ions and alginate chains not only enables the tuning of the mechanical properties of the tough organohydrogels in bulk, but also allows controlling of the stiffness locally when combined with various surface patterning techniques. As shown in Figure 1a, the tough organohydrogels can be encoded with spatially distributed mechanical properties, that is, anisotropic elements with different elastic moduli, by the use of a paper-based transfer patterning technique. The stiff anisotropic structures are utilized as elements for programmed guiding of shape transformation of organohydrogels by exerting (or relaxing) mechanical stress on (or from) the patterned organohydrogels. Thus, we applied this strategy to the programmed control of shape morphing of tough organohydrogels, with a particular focus on demonstrating the following two unique features: anti-freezing and non-drying.

**Figure 2:**
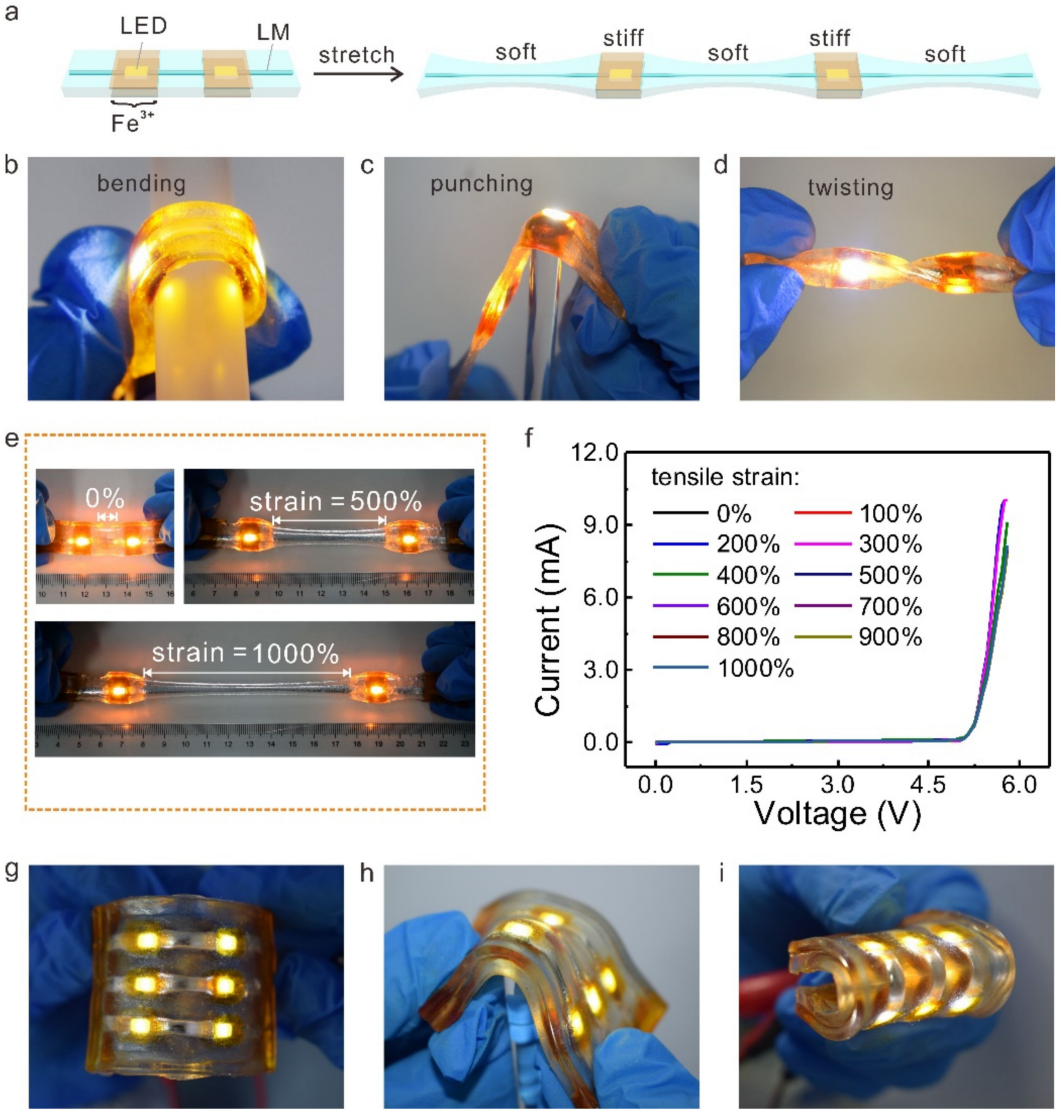
Conformal packaging of “island–bridge” stretchable electronics. (**a**) Scheme of the soft–stiff–soft–stiff–soft “island–bridge” configuration enabled by Fe (III) stiffening and stretching of the soft (not iron (III)-treated) component. (**b**–**d**) Photographs showing the resistance of the “island–bridge” circuit against various mechanical stresses, such as (**b**) bending, (**c**) punching, and (**d**) twisting. (**e**) The “island–bridge” circuit before and during stretching (500% and 1000% tensile strain). (**f**) I–V characteristic curves of the “island–bridge” circuits at different tensile strains. (**g**–**i**) Array of LEDs in a flexible circuit at various mechanical deformations, which are locally stiffened with Fe (III) at the LED locations.

The first unique feature of the present organohydrogels is that programmable shape morphing can be conducted at subzero temperatures. Traditionally, hydrogels are easily frozen at subzero temperatures due to significant water content. As such, the mechanical properties dramatically change, leading to failure of the programmable shape morphing. In contrast, organohydrogels can undergo shape morphing without being frozen, owing to their extremely high freezing tolerance. As proof-of-concept, shape morphing experiments of hydrogels and organohydrogels were conducted at a temperature of approx. −50 °C. Indeed, the hydrogels freeze quickly when exposed to such a low temperature, while the organohydrogels maintain their soft and stretchable state (Figure 2b). The flexibility of the organohydrogel at these temperatures is assigned to the partial replacement of water molecules with glycerol molecules. Glycerol is a common cryoprotectant [44], which reduces the freezing temperature in glycerol/water mixtures and inhibits ice formation [45]. Indeed, in differential scanning calorimetry (DSC) thermograms, no freezing peak for the organohydrogel and the stiffened hydrogel was observed in the range between −80 °C and 25 °C, as shown in Figure S6. The reduced amount of water in the gel was corroborated with thermogravimetric analysis of the different gels (see Figure S7).

After relaxing of a pre-applied tensile strain, the frozen hydrogels maintain their elongation state due to their frozen state. In contrast, the organohydrogel quickly folds into a 3D structure when relaxed. Furthermore, the folding of organohydrogels is reversible by repeating the stretching–relaxing cycle (Supplementary Materials, Video S1). The hydrogels, however, are non-stretchable and brittle, enabling rupture and failure during mechanical stress, such as stretching, twisting, or folding. The different behavior of the hydrogel and the organohydrogel can be explained by the formation of crystals of water in hydrogels, while the organohydrogels do not freeze down to a temperature of approximately −50 °C. Therefore, the gel structure remains mechanically durable, flexible, and stretchable. As such, organohydrogels can fold into 3D structures when the tensile strain is relieved. Figure 1c illustrates that it is possible to morph an organohydrogel into a more complex 3D shape, signifying the great potential of this method.

The second feature of our approach is that the folded structures can be maintained for an extended period, even under arid or vacuum conditions. Although some studies employ water moisture to trigger the shape morphing of hydrogels, the dehydration of the folded structures can cause the failure of the shape morphing system after a long period. As such, the application of these shape-morphing systems is limited to environments with high humidity and moderate temperatures. In the present study, owing to the high drying tolerance, the organohydrogels can not only undergo shape morphing under arid conditions, but also, more significantly, keep their folded shapes even after vacuum freeze-drying and after storage under arid conditions for an extended period. To prove this drying resistance, the organohydrogel and the hydrogel were exposed to a dry environment. Figure S8 shows the weight retention of the hydrogel and organohydrogel when exposed to a relative humidity of 10%. The weight of the hydrogel dwindles in 4 days by 50%, signifying extensive evaporation. In contrast, the organohydrogel virtually maintained its weight during exposure to these dry conditions. Figure 1d shows the folding of an organohydrogel into a tube-shaped structure before and after vacuum freeze-drying, further signifying the drying tolerance. Figure 1f–h shows various folded structures before, during, and after storage up to 70 days, signifying that the long-term preservation of these shape-morphed organohydrogel structures guarantees a practical application of the shape morphing in arid conditions. In contrast, shapes made out of conventional shape-morphing hydrogels are subject to dehydration, get stiff and brittle, and lose their programmed shape (Figure 1e).

Not limited to shape morphing, the anti-freezing and non-drying organohydrogels with spatially defined mechanical properties encoded within one material are ready to be applied as a promising material for conformal packaging of stretchable electronics. To date, there are mainly two strategies to realize stretchable electronics: (1) structures and (2) materials that can be stretched [2,46,47,48]. Notably, most stretchable electronics typically consist of non-stretchable, stiff, and brittle electronic components, such as LED lamps. Traditional packaging of the rigid units with a soft elastomer would cause delamination due to mechanical mismatch, attributed to different elastic moduli. Furthermore, the mechanical stress applied to the elastomer is directly transferred to the rigid and brittle electronic components, which is harmful to the performance of the electronic device and may lead to tears, cracks, or disconnection. Indeed, an unprotected circuit shows crack formation at the interface between the rigid electronics and the soft part upon application of a tensile strain of 130%, as shown in Figure S9.

To address this issue, we demonstrate the utility of the current localized stiffening strategy and technology platform with the organohydrogels by fabricating “island–bridge” stretchable circuits, which consist of soft liquid metal-based conductors and the incorporated arrays of rigid LED lamps. As shown in Figure 2a, the rigid “island” structures possess a much higher elastic modulus, which is barely stretched for mounting rigid electronic components. The soft “bridge” structures, however, are highly stretchable to act as or to allow the incorporation of soft components such as room-temperature liquid metal conductors (galinstan) [49,50]. As such, the integration of the rigid and soft components within one patch of tough organohydrogels is readily achieved. As proof-of-concept, arrays of rigid LED lamps were packaged and interconnected with soft liquid-metal conductive channels. Indeed, the as-made “island–bridge” circuits exhibit extremely high flexibility and stretchability, which can be verified by conducting various mechanical deformations, including bending, twisting, punching, and stretching (Figure 2b–e). The optical images clearly show that the regions for mounting the LED lamps and their physical shapes are well maintained despite various mechanical deformations. In contrast, the regions of the soft liquid metal-based conductive paths are easily stretched and deformed to absorb the applied stress (Supplementary Materials, Video S2). The characteristic I–V curves of the circuits at different tensile strains ranging from 0 to 1000% further indicate the stability of the integration of the circuits (Figure 2f). The present rigid “island” and soft “bridge” structure can be considered as a combination of the abovementioned structure and material strategies for stretchable electronics. Therefore, the whole circuits can be considered to be intrinsically stretchable with site-specific stiffened structures to allow the conformal packaging of non-stretchable electronic components.

**Figure 3:**
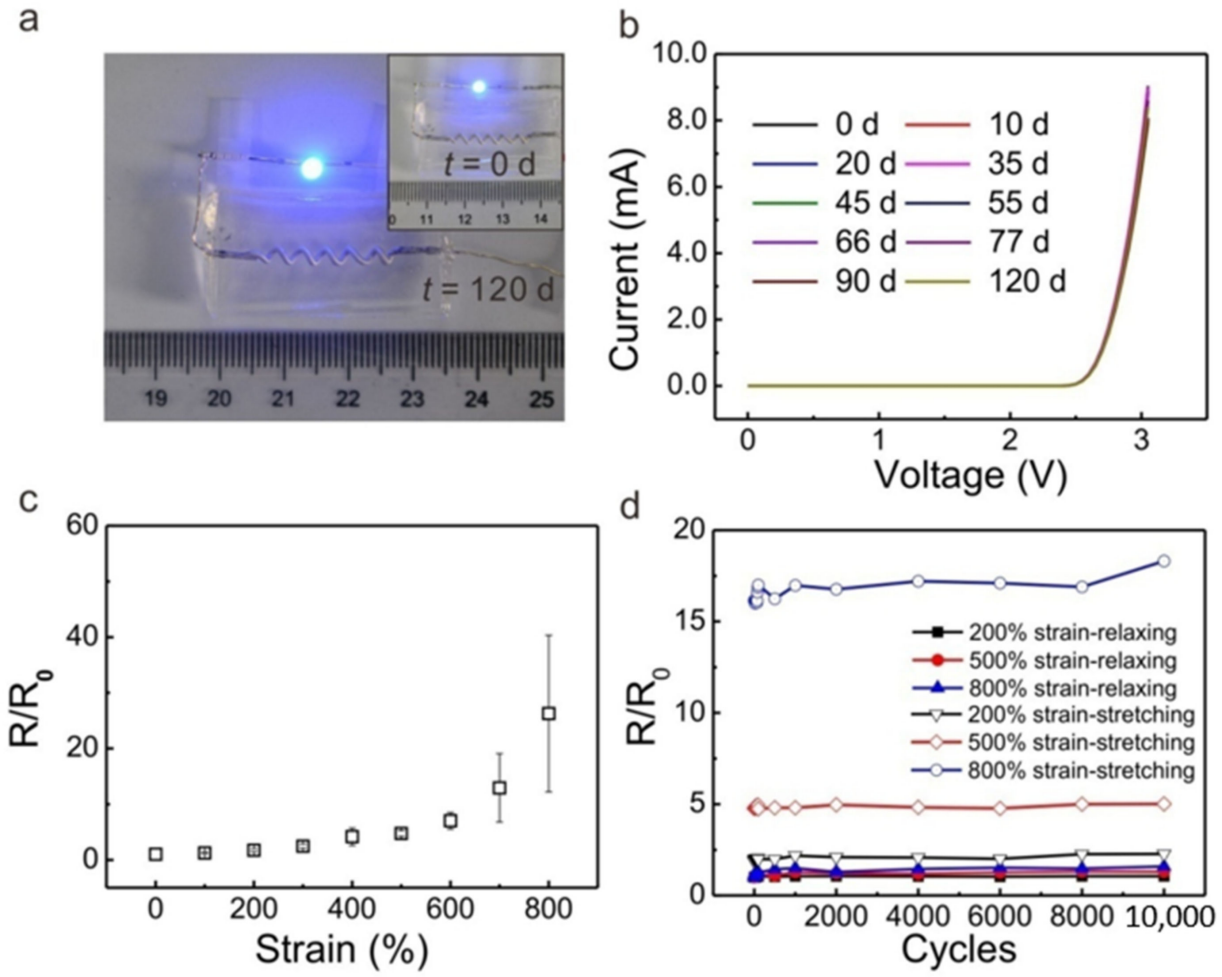
Durability of liquid-metal organohydrogel circuits and their electrical performance. (**a**) Photographs of the as-prepared (inset) and aged (120 days, 50% humidity) organohydrogel circuit. (**b**) Characteristic I–V curve of the as-prepared organohydrogel circuit and after storage for up to 120 days. (**c**) Ratio of the resistance (R/R_0_), where R is the resistance at applied tensile strain and R_0_ denotes for the resistance without tensile strain. (**d**) Fatigue test: ratio of the resistance of the circuit from (**a**) during stretching and relaxing for up to 10,000 cycles.

In addition, the high drying tolerance of the insulating tough organohydrogels enables long-term and practical use of the as-made stretchable electronics under harmful and challenging conditions. Significantly, owing to the solvent displacement, the ionic conductive hydrogels change to insulating organohydrogels (Figure S10). Therefore, the features of the tough organohydrogels mentioned above have demonstrated their excellent capabilities to serve as promising materials for packaging of stretchable electronics. As such, we conducted the characterization of an LED-incorporated liquid-metal organohydrogel-based circuit for a storage time of up to 4 months (Figure 3a–b). The organohydrogel circuit showed stable performance in the observed time frame. In contrast to the extended period of stable performance of the organohydrogels, traditional hydrogels become dry within two days. The organohydrogels exhibit not only extended drying tolerance, but also fatigue resistance and excellent electrical performance upon tensile strain. Fatigue test of the change in resistance of an organohydrogel circuit based on liquid metal was conducted at different tensile strains up to 800% for 10,000 cycles. As shown in Figure 3c–d, the ratio of resistance (R/R_0_), where R is the resistance at applied tensile strain and R_0_ denotes for the resistance without tensile strain, increases slowly from 1.0 to 7.0 ± 1.5 between 0 and 600% tensile strain, respectively. Afterward, the increase is steeper with a ratio of 26 ± 14 at 800% tensile strain. The incline in resistance of the circuit at increased tensile strain is attributed to the thinning of the liquid-metal conductor. However, the fatigue test in Figure 3d shows that the change in resistance is reversible upon release of the tensile strain, while the resistance is virtually constant upon increasing the number of load/unload cycles.

## 3. Conclusions

In conclusion, we have developed a reliable and straightforward strategy for the rational design of anti-freezing, non-drying, localized stiffening, and shape-morphing organohydrogels. Not limited to shape morphing, complex “island–bridge” structures were demonstrated for stretchable electronics that can work for a long period under challenging conditions. To the best of our knowledge, this study for the first time reports several significant findings, such as localized stiffening of tough organohydrogels, shape morphing of gel-based materials at subzero temperatures and under arid or even vacuum freeze-drying conditions, and the conformal packaging of hard and soft electronic components with locally stiffened tough organohydrogels. Although the present study demonstrates the design of a programmable shape-morphing system with tough organohydrogels and the utility of this material for packaging of functional components with different elastic moduli, the core strategy and the as-developed technology platform can be employed for a wide range of applications with extended conditions in challenging environments. For example, our strategy has implications towards the bio-inspired design of human–machine/machine–machine interfaces [33], soft robotics [51], sensors [52], soft electronics [53], and actuators [20,22], with a particular focus on locally tunable mechanical properties and working under more challenging environments.

## 4. Materials and Methods

Materials: Sodium alginate was purchased from Aladdin (Shanghai, China). Acrylamide, ammonium persulphate, calcium sulfate dihydrate, glycerol, ferric chloride hexahydrate (FeCl_3_·6H_2_O), and ammonium sulfate were purchased from Macklin. N,N’-methylenebisacrylamide and N,N,N’,N’-tetramethylethylenediamine were purchased from Sigma-Aldrich (St. Louis, USA). Advantec No. 2 filter paper was used for the ion transfer printing. Galinstan (gallium (~68.5%)–indium (~21.5%)–tin (~10%), 6.440 kg/L) alloy was purchased from Wochang (Dongguan, China). Deionized (DI) water (18 MΩ·cm) was used unless stated otherwise.

Synthesis of Ca-alginate/PAAm tough organohydrogels: First, sodium alginate (0.25 g) was dissolved in 12.5 mL DI water and agitated by ultrasonication until sodium alginate was completely dissolved. Second, 2.00 g acrylamide was added to the prepared solution and agitated ultrasonically for 2 h. Third, 96 μL of 0.025 g/mL N,N’-methylenebisacrylamide (MBAA, crosslinking agent) aqueous solution and 10 μL of N,N,N’,N’-tetramethylethylenediamine (TEMED, crosslinking accelerator) were added. Subsequently, the mixture was stirred for 30 min. Afterward, 4 mL 0.033 g/mL calcium sulfate dihydrate solution was added at an injection speed of 0.08 mL/min and the resulting solution degassed. Finally, 60 μL 0.1 g/mL ammonium persulphate solution (APS, photoinitiator) was added. The prepared solution was poured into a plastic mold and cured with UV light (254 nm, 25 W, model: ZF-5, company: Shanghai Jiapeng, Shanghai, China) for 40 min at a temperature of 40 °C. Subsequently, the solidified mixture was stored for stabilization for 1 day. Then, the tough glycerol-based organohydrogels were prepared by the solvent displacement method. Briefly, the Ca-alginate/PAAm hydrogels were immersed in glycerol for 6 h. Afterward, the soaked hydrogels were removed from the container and excess solvent on hydrogels was dabbed off with a filter paper.

Preparation of liquid-metal circuits with 3D spiral structure based on tough organohydrogels: The prepared solution was poured into a mold, in which a silver helical spring (wire diameter: 0.9 mm, outer diameter: 3 mm, pitch gauge: 3.5 mm) was placed; then, it was covered with a polyimide (PI) film and irradiated with ultraviolet light (wavelength of 254 nm, 25 W) for 40 min while being held at 40 °C. After curing of the hydrogel, it was removed from the mold. Subsequently, the spring was carefully removed from the hydrogel, leaving a 3D spiral channel in the hydrogel. Galinstan was injected with a syringe into the channel. Finally, the tough organohydrogel-based circuit was obtained by the solvent displacement method (6 h immersion time).

Preparation of “island–bridge” stretchable electronics and programmable shape morphing at subzero temperatures: The prepared solution was poured into a plastic mold and cured with UV light (254 nm, 25 W) for 30 min at a temperature of 40 °C. The resulting semi-cured hydrogel was stored for stabilization for 30 min. Subsequently, an LED circuit with galinstan wire as a conductor was placed on the surface of the semi-cured hydrogel. Then, a certain amount of the prepared solution was poured into the mold to package the LED circuit and cured with UV light (254 nm, 25 W) for 40 min at a temperature of 40 °C. The packaged circuit was stored one day for stabilization. Afterward, the hydrogel circuit was immersed in glycerol for solvent displacement (6 h). The soaked hydrogel circuit was removed from the container and excess solvent on it was removed with dry filter paper. Then, the paper was cut into the desired shapes and soaked in freshly prepared 1 M ferric (III) chloride solution. Excess solution from the filter paper was removed with a dry filter paper. Then, the filter paper loaded with Fe (III) ions was brought into contact with the tough organohydrogel for 5 min. For achieving programmable shape morphing of the tough organohydrogel at −50 °C, the tough organohydrogel was pre-stretched at a tensile strain of 100% prior to patterning with Fe (III) ion solution. Afterward, the filter paper was removed. Next, 20 min after the removal of the filter paper, the tensile strain was removed. At this point, the organohydrogel starts deforming, for example, it bent or even folded toward the untreated organohydrogel side at −50°C.

Mechanical characterization of organohydrogels: Tensile tests of the Fe(III)-treated Ca-alginate/PAAm organohydrogels were carried out by using a CMT6103 electronic universal testing machine (SANS, Shenzhen, China). The cuboid-shaped gels were prepared in the following dimensions: total length = 40 ± 0.5 mm, effective length = 19 ± 3 mm, width = 17 ± 1.5 mm, and thickness = 2 ± 0.4 mm. Both ends of the sample were fixed with clamps. The samples were stretched at a constant speed of 60 mm/min. The force–length curves and the stress–strain curves were obtained from the force–displacement curves. The elastic modulus (Young’s modulus) was determined from the stress–strain curve by the average slope (between 0 and 15% tensile strain).

Measurement of electric performance: Organohydrogel circuits were stretched repeatedly by using a computer-controlled moving stage (TSA1000-B, Zolix, Beijing, China). Stretching speed was set to 20 mm per second. The liquid metal circuits with 3D spiral structure based on organohydrogels had the following dimensions: total length = 34.5 ± 0.5 mm, effective length = 13 ± 2 mm, width = 16.5 ± 0.5 mm, and thickness = 4.3 ± 0.2 mm. The resistance under tensile stress and that in the relaxed state were measured separately. The resistance of the organohydrogel circuit was measured with a Keithley 2400 sourcemeter (Tektronix, Beaverton, OR, USA) by using 4-point probe method. I–V characterization of the LED circuits was conducted by using a Keithley 2400 sourcemeter (Tektronix, Beaverton, OR, USA) controlled via a GPIB remote control. Voltage sweep was conducted between 0 and 5 V at rate of 0.05 V/s (current limit 0.01A).

Conductivity Measurement: Conductivity was measured by using electrochemical impedance spectroscopy (EIS). The AC signals of the impedance spectroscopy measurements were produced by potentiostatic EIS with a VersaSTAT 4 (Princeton Applied Research, Oak Ridge, TN, USA), with a frequency sweep ranging from 1000 kHz to 0.1Hz (10 points per decade). The amplitude of the alternating signal on the zero-volt bias was 10 mV.

Characterization: The scanning electron microscopy (SEM) imaging and energy-dispersive X-ray spectroscopy (EDS) mapping of the Ca-alginate/PAAm tough hydrogel and the Fe^3+^-treated hydrogel were performed with a high-resolution scanning electron microscope (APREO S, ThermoFisher Scientific, Waltham, MA, USA). The samples for SEM imaging and EDS mapping were prepared by freeze-drying under vacuum. The Fourier transform infrared (FT-IR) spectra were recorded on an IR Affinity-1 (Shi-madzu, Kyoto, Japan) spectrometer. The thermal analysis was performed using a differential scanning calorimeter (DSC, DSC-200F3 NETZSCH, Selb, Germany) in the temperature range from −80 °C to 25 °C at a rate of 5 °C min^−1^ under a nitrogen atmosphere (flow rate: 50 mL min^−1^). The thermogravimetric analysis was performed with a simultaneous thermal analyzer (TG-DTA, STA409PC, NETZSCH, Selb, Germany) in the range from room temperature up to 800 °C at a heating rate of 10 °C/min.

## Data Availability

The data that support the findings of this study are provided as source data with this paper or are included in the Supplementary Materials. Further data are available from the corresponding authors upon request.

## Supplementary Materials

The following supporting information can be downloaded at https://www.mdpi.com/article/10.3390/gels8060331/s1. Figure S1: Schematic illustration of the double network (DN) hydrogel (top left) and its corresponding constituents alginate gel (crosslinked by Ca^2+^, right) and crosslinked polyacrylamide (bottom left, blue). Furthermore, the crosslink between the polyacrylamide and alginate gel is illustrated (bottom middle); Figure S2: ATR-FTIR (attenuated total reflection–Fourier transform infrared) spectra of the original DN hydrogel (Ca-alginate/polyacrylamide), the glycerol-treated gel (denoted organohydrogel), and the iron (III)-treated organohydrogel (denoted stiffened organohydrogel) [54,55]; Figure S3: (a) The stress–strain curves and (b) the elastic modulus of the Fe(III)-stiffened Ca-alginate/PAAm organohydrogels prepared with soaking times ranging from 0 to 60 min; Figure S4: (a) Stress–strain curves of the Fe^3+^-treated (soaking time 5 min) organohydrogel dependent on the environmental humidity. Prior to measurement, the treated organohydrogel was exposed to the environment with designated humidity for 24 h (in a humidity chamber). The test was conducted at room temperature at a speed of 20 mm/min. (b) Elastic modulus of the iron (III)-treated organohydrogel exposed to varying humidity, corresponding to the graph shown in (a); Figure S5: (a) SEM micrographs of the DN hydrogel and the stiffened hydrogel together with the two-dimensional energy dispersive X-ray spectroscopy (EDS) maps showing the elemental distribution of C, N, O, Ca, Na, Fe, and Cl in the gels. (b) Column diagram signifying the atomic percentage of the aforementioned elements in the hydrogel and stiffened hydrogel; Figure S6: DSC thermogram of the DN hydrogel (red), organohydrogel (black), and Fe^3+^-treated organohydrogel in the range from −80 to 25 °C at a heating rate of 5 °C/min. The solid line denotes the cooling run, while the dashed line denotes the heating run; Figure S7: Thermogravimetric analysis (TGA) of (a) the DN hydrogel, (c) the corresponding organohydrogel, (e) and the Fe^3+^-treated organohydrogel in the range from room temperature up to 800 °C at a heating rate of 10 °C/min. Below each measurement, the derivative gravimetric curve is depicted: (b) DN hydrogel, (d) organohydrogel, and (f) stiffened organohydrogel; Figure S8: Weight retention of the organohydrogel and hydrogel upon storage in a desiccator at room temperature and 10% relative humidity; Figure S9: Demonstration of crack formation occurring for stiff electronics packaged in the soft organohydrogel. Upon straining the composite with a tensile strain of 130 %, a tear of the stiff conductor was observed; Figure S10: The conductivity of the hydrogel and organohydrogel prepared at different solvent displacement times from 0.5 to 6.0 h; Video S1: A tough hydrogel and tough organohydrogel were cooled to a temperature of approximately −50 °C. The tough hydrogel freezes quickly, while the tough organohydrogel remains unfrozen. After relaxing of a pre-applied tensile strain, the frozen tough hydrogels retains its elongated state. In contrast, the tough organohydrogel quickly folds into a 3D structure. Furthermore, we show that the folding of the tough organohydrogel is reversible by repeating the stretching–relaxing cycle; Video S2: The video shows that “island–bridge” stretchable electronics can be stretched up to 1000% and that the tensile strain can be cycled for many times.
