# Supplementary material for: Anti-Freezing, Non-Drying, Localized Stiffening, and Shape-Morphing Organohydrogels
Source: Gels. 2022 May 25;8(6):331. doi: 10.3390/gels8060331 (PMC9222875; doi:10.3390/gels8060331)
Supplement: Supplementary file 1 [file gels-08-00331-s001.zip › gels-1708685-supplementary.pdf]

gels

MDPI

Article

Anti-Freezing, Non-Drying, Localized Stiffening, and Shape-Morphing Organohydrogels

Jiayan Shen [1], Shutong Du [1], Ziyao Xu [1], Tiansheng Gan [1], Stephan Handschuh-Wang [1] and Xueli Zhang [1,*]

[1] College of Chemistry and Environmental Engineering, Shenzhen University, Shenzhen, China; 2170225620@email.szu.edu.cn (J.S.); 2060221013@email.szu.edu.cn (S.D.); 1910223058@email.szu.edu.cn (Z.X.); gantiansheng@szu.edu.cn (T.G.); stephan@szu.edu.cn (S.H.-W.);

* Correspondence: xlzhang@szu.edu.cn; Tel.: +86-755-26557377

Supporting Information

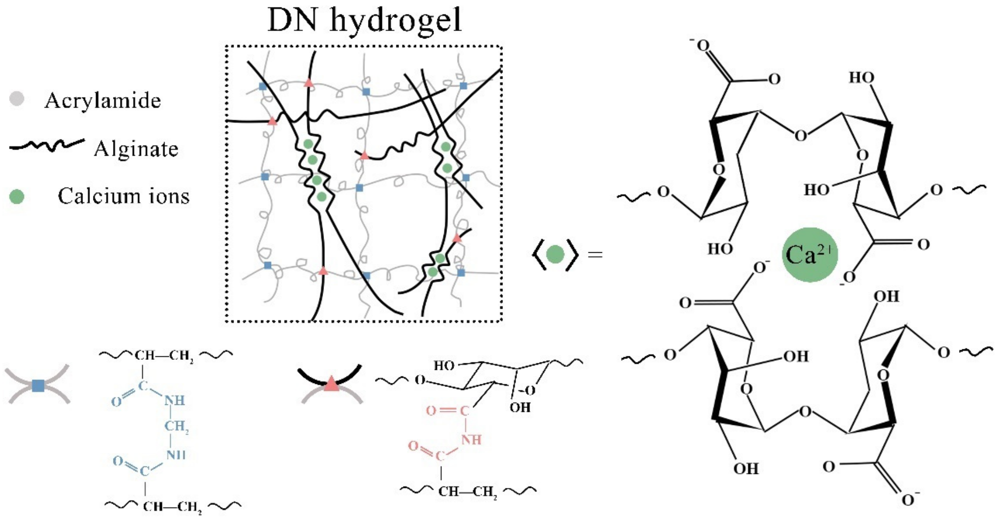

Figure S1. Schematic illustration of the double network (DN) hydrogel (top left) and its corresponding constituents alginate gel (crosslinked by Ca^{2+}, right) and crosslinked polyacrylamide (bottom left, blue). Furthermore, the crosslink between the polyacrylamide and alginate gel is illustrated (bottom middle).

The FTIR spectra of the three gels (DN hydrogel, glycerol-based organohydrogel, and stiffened gel) are shown in Figure S2. The alginate in the DN gel exhibits a broad peak near 3340 cm^{-1}, indicative for O–H stretching, a sharp peak at 1600 cm^{-1} assigned to asymmetric COO– stretching, and two peaks at 1420 and 1320 cm^{-1} for C–H deformation with secondary alcohols. Furthermore, three peaks at 1120, 1090, and 1030 cm^{-1} were found for asymmetric C–O–C stretching, C–O stretching in CH–OH structure, and symmetric C–O stretching in C-O-C structure, respectively. Similarly, in the hydrogel, the bands for polyacrylamide were found. The gel exhibited bands at 3360 cm^{-1} and 3200 cm^{-1}, which correspond to a stretching vibration of N–H. The band at 1660 cm^{-1} is indicative for C=O stretching. The bands at 1600 cm^{-1} (N–H deformation for primary amine), 1450 cm^{-1} (CH_2 in-plane scissoring, very minor), 1420 cm^{-1} (C–N stretching for primary amide), 1350 cm^{-1} (C–H deformation), and 1120 cm^{-1} (NH_2 in-plane rocking) were also detected. The spectrum of the DN gel features a peak at 1280 cm^{-1} for C–N stretching of secondary amide, which was formed during polymerization. This result indicates chemical bond formation between -NH_2 groups of polyacrylamide and carboxyl groups of alginate.

The organohydrogel features similar absorption bands to the DN hydrogel. In addition, it features the bands stemming from the glycerol, and due to the presence of an abundance of glycerol in the gel, these bands are stronger than that of the gel. Novel bands are as follows. The band at 850 cm^{-1} denotes C–C rocking, the band at 920 cm^{-1} denotes C–OH stretching, and the band at 1100 cm^{-1} denotes the C–O stretching vibration. Further new bands are at 1200 cm^{-1} (C–H wagging), 1450 cm^{-1} (C–H bending), and 2875 cm^{-1} (symmetric C–H stretching). [54] Slight shifts in band location (10 cm^{-1}) might be due to association effects, i.e., association with glycerol or ions. [55] We assume that the reduction in absorption band at 1670 cm^{-1} is related to the association of the iron (III) with the O of the C=O.

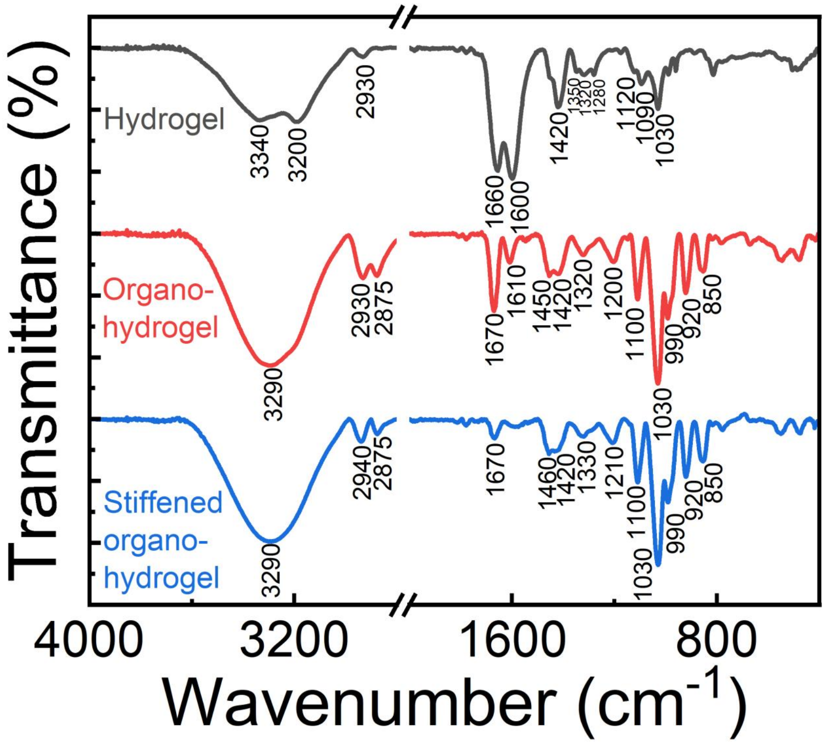

Figure S2. ATR-FTIR (attenuated total reflection–Fourier transform infrared) spectra of the original DN hydrogel (Ca-alginate/polyacrylamide), the glycerol-treated gel (denoted organohydrogel), and the iron (III)-treated organohydrogel (denoted stiffened organohydrogel).

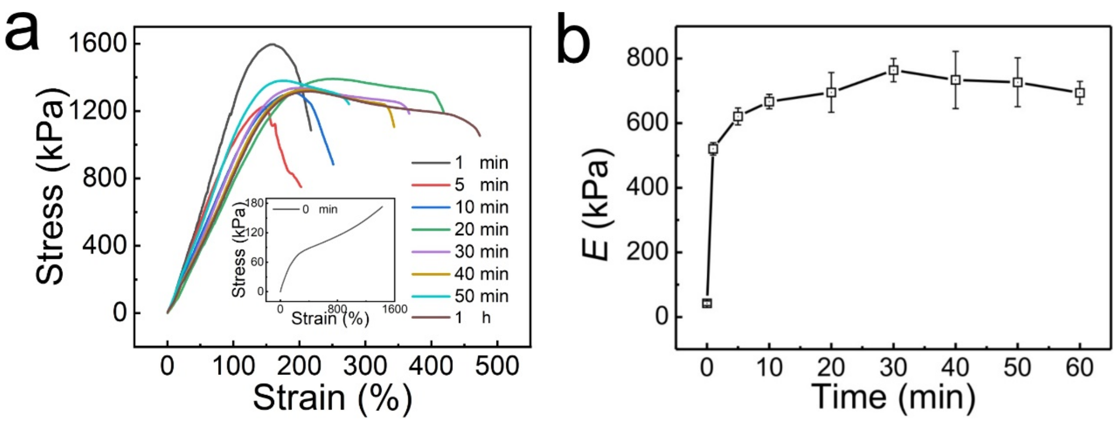

Figure S3. (**a**) The stress–strain curves and (**b**) the elastic modulus of the Fe(III)-stiffened Ca-alginate/PAAm organohydrogels prepared with soaking times ranging from 0 to 60 min.

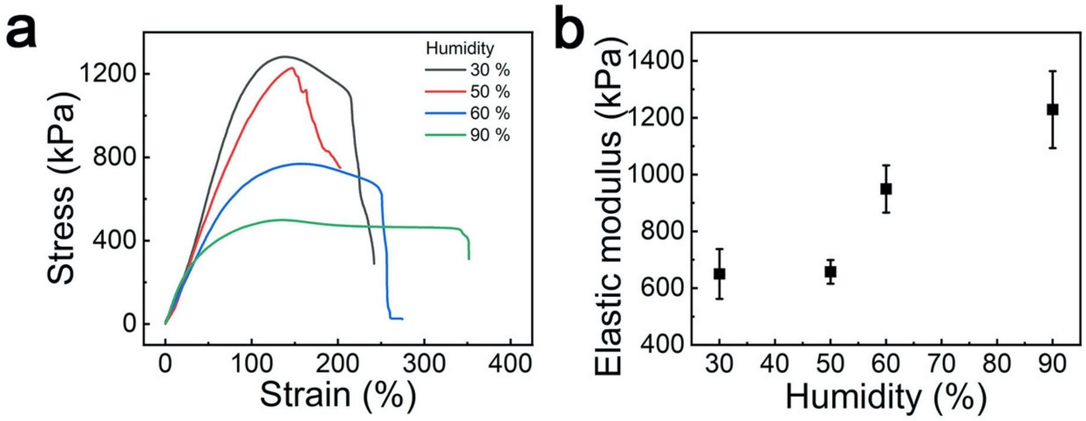

Figure S4. (**a**) Stress–strain curves of the Fe^{3+}-treated (soaking time 5 min) organohydrogel dependent on the environmental humidity. Prior to measurement, the treated organohydrogel was exposed to the environment with designated humidity for 24 h (in a humidity chamber). The test was conducted at room temperature at a speed of 20 mm/min. (**b**) Elastic modulus of the iron (III)-treated organohydrogel exposed to varying humidity, corresponding to the graph shown in (**a**).

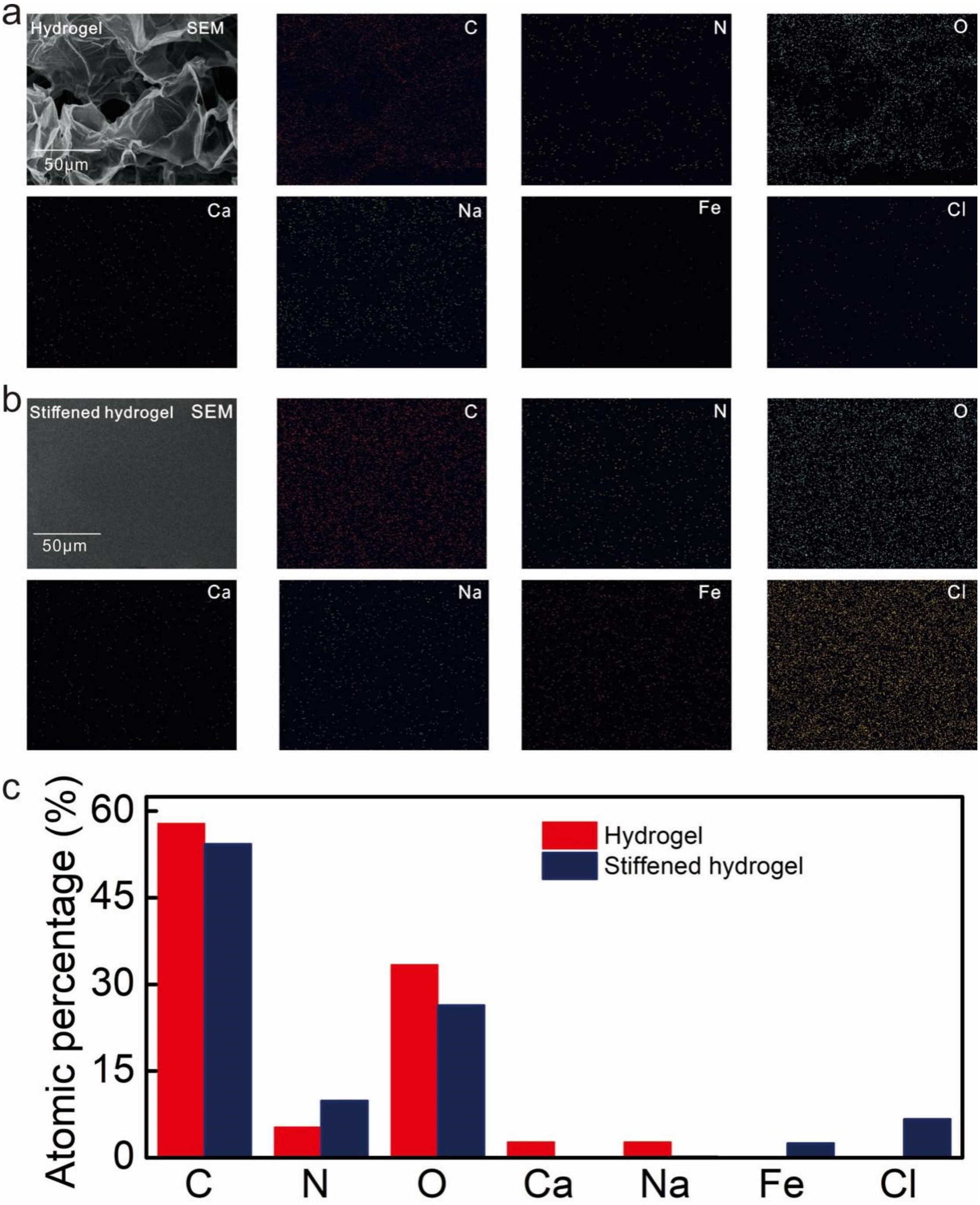

Figure S5. (**a**) SEM micrographs of the DN hydrogel and the stiffened hydrogel together with the two-dimensional energy dispersive X-ray spectroscopy (EDS) maps showing the elemental distribution of C, N, O, Ca, Na, Fe, and Cl in the gels. (**b**) Column diagram signifying the atomic percentage of the aforementioned elements in the hydrogel and stiffened hydrogel.

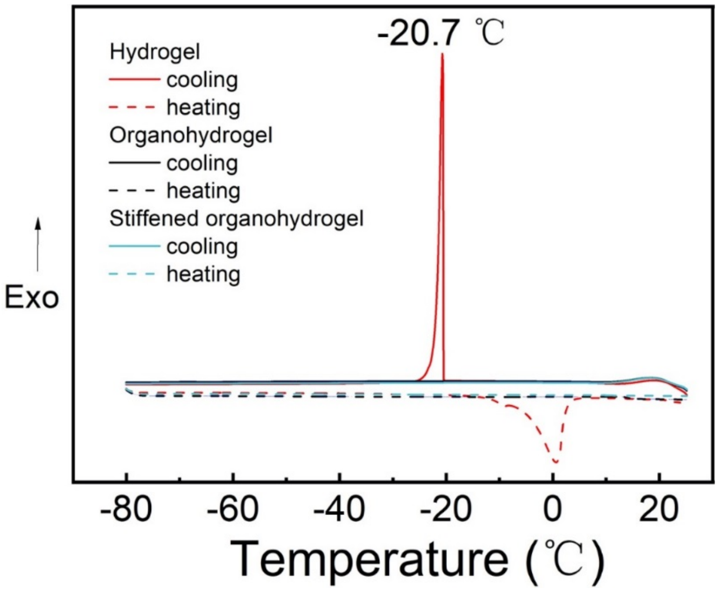

Figure S6. DSC thermogram of the DN hydrogel (red), organohydrogel (black), and Fe^{3+}-treated organohydrogel in the range from −80 to 25 °C at a heating rate of 5 °C/min. The solid line denotes the cooling run, while the dashed line denotes the heating run.

The thermogravimetric analysis of the hydrogel shows 3 derivatives (steps). The first at around 100 °C is related to the evaporation of free water. We assign the second step at around 200 °C to evaporation of bound water (bound by the polymer). The last step is attributed to the decomposition of the polymers (300–400 °C). For the organohydrogel, less water evaporates at 100 °C due to thorough displacement of water by glycerol. At 200 °C, a high weight loss is observed due to evaporation of glycerol. Decomposition of the polymer is observed at 300–400 °C. A similar shape of the thermogram is seen for the stiffened organohydrogel. However, the water evaporation step is bigger due to the treatment with aqueous iron (III) solution.

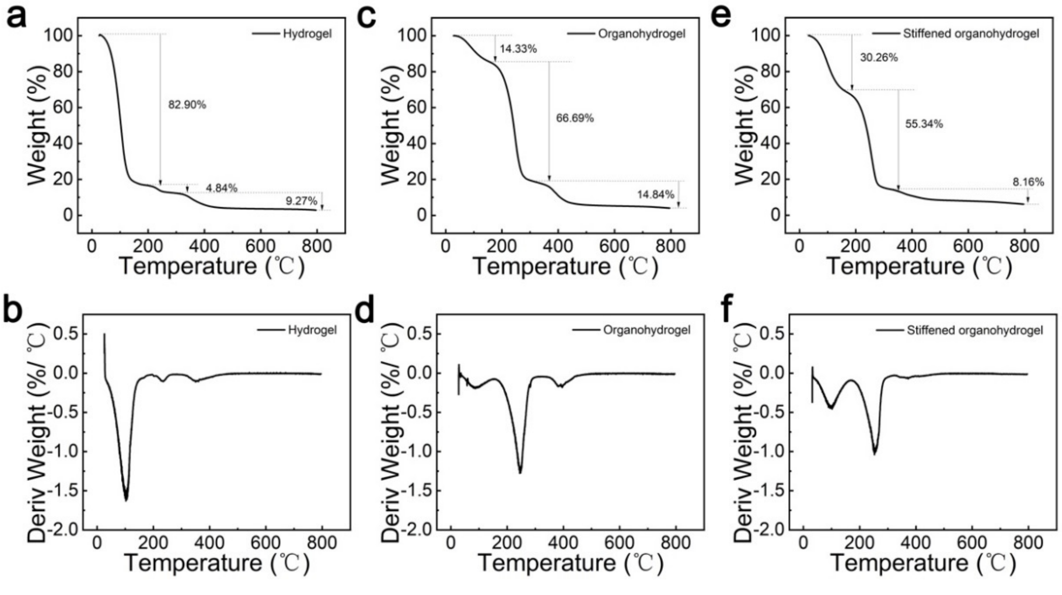

Figure S7. Thermogravimetric analysis (TGA) of (**a**) the DN hydrogel, (**c**) the corresponding organohydrogel, (**e**) and the Fe^{3+}-treated organohydrogel in the range from room temperature up to 800 °C at a heating rate of 10 °C/min. Below each measurement, the derivative gravimetric curve is depicted: (**b**) DN hydrogel, (**d**) organohydrogel, and (**f**) stiffened organohydrogel.

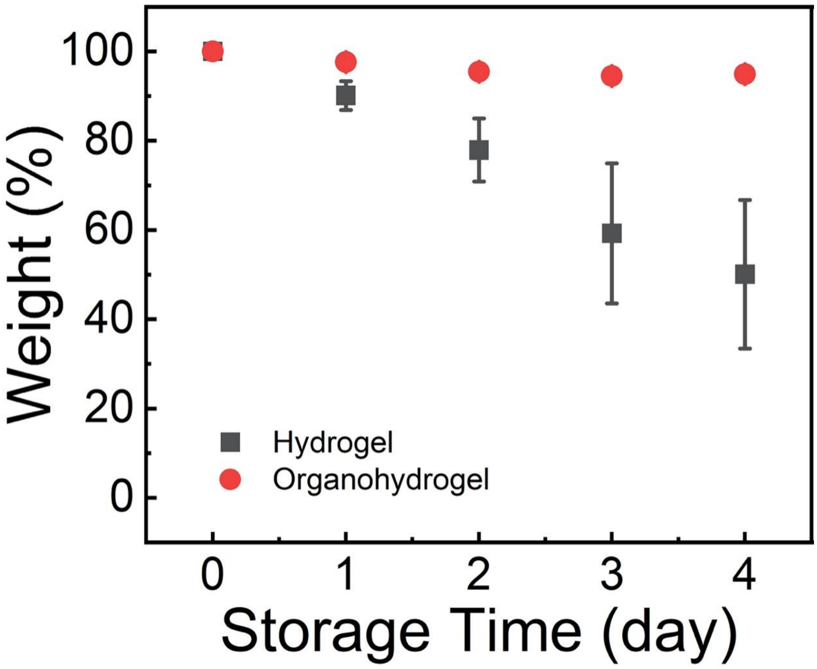

Figure S8. Weight retention of the organohydrogel and hydrogel upon storage in a desiccator at room temperature and 10% relative humidity.

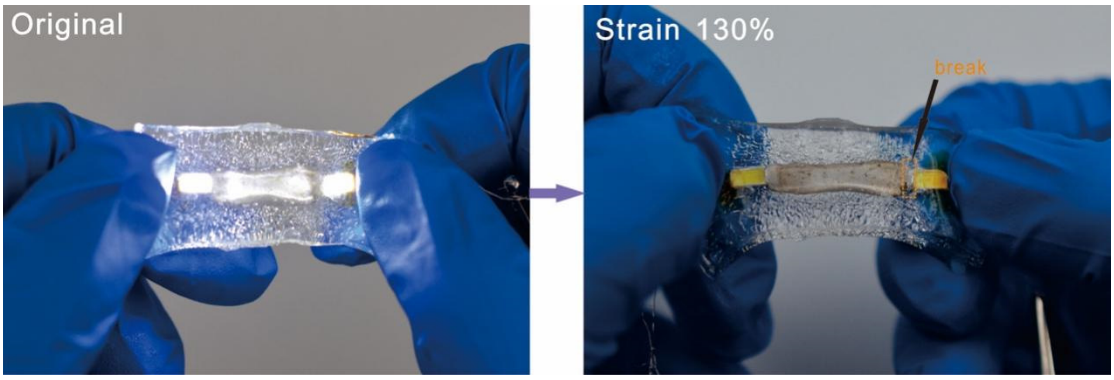

Figure S9. Demonstration of crack formation occurring for stiff electronics packaged in the soft organohydrogel. Upon straining the composite with a tensile strain of 130 %, a tear of the stiff conductor was observed.

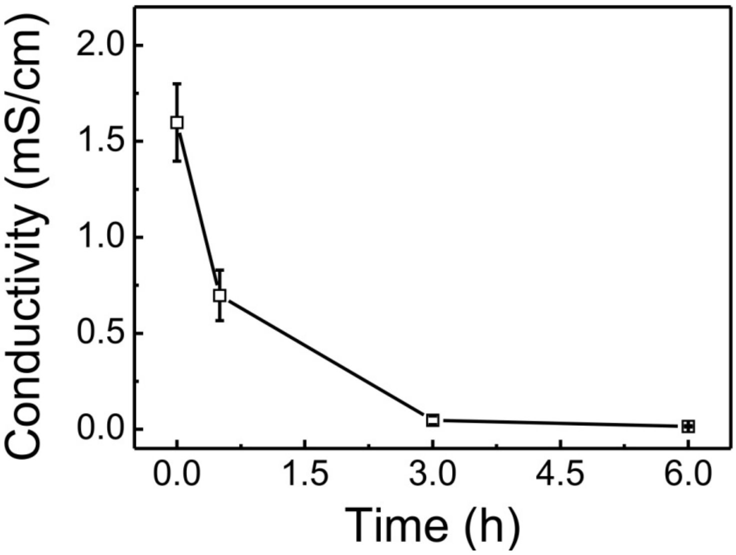

Figure S10. The conductivity of the hydrogel and organohydrogel prepared at different solvent displacement times from 0.5 to 6.0 h.

Video S1: A tough hydrogel and tough organohydrogel were cooled to a temperature of approximately −50 °C. The tough hydrogel freezes quickly, while the tough organohy-drogel remains unfrozen. After relaxing of a pre-applied tensile strain, the frozen tough hydrogels retains its elongated state. In contrast, the tough organohydrogel quickly folds into a 3D structure. Furthermore, we show that the folding of the tough organohydrogel is reversible by repeating the stretching–relaxing cycle.

Video S2: The video shows that "island–bridge" stretchable electronics can be stretched up to 1000% and that the tensile strain can be cycled for many times.
